# Protocol for lentivirus-mediated delivery of genes to study neurogenesis and cognitive function in adult rodents

**DOI:** 10.1016/j.xpro.2023.102761

**Published:** 2023-12-02

**Authors:** Li Xu, Changyong Tang

**Affiliations:** 1 Department of Neurology, the Third Affiliated Hospital of SUN Yat-sen University, 600 Tianhe Road, Guangzhou, Guangdong 510630, P.R. China

**Keywords:** Cognitive Neuroscience, Behavior

## Abstract

Adult neurogenesis leads to the generation of functional neurons from neural stem cells, whereas impairment of adult hippocampal neurogenesis contributes to the pathophysiology of cognitive symptoms in neurodegenerative and neuropsychiatric diseases. Here, we present a protocol for a direct hippocampal injection of lentivirus-delivered gene in adult rodents to study the specific molecular mechanism underlying adult neurogenesis, including lentivirus packaging and stereotaxic injection, EdU and BrdU injections, tissue immunostaining and imaging analysis, and cognitive testing.

For complete details on the use and execution of this protocol, please refer to Li et al. (2023).^1^

## Highlights

• Step-by-step guide for lentivirus production
• Protocol for stereotactic lentiviral injection into the dentate gyrus of rodent hippocampus
• Evaluation assays for neurogenesis and cognitive function *in vivo*

## Abstract (editor highlights)

Publisher’s note: Undertaking any experimental protocol requires adherence to local institutional guidelines for laboratory safety and ethics.

## Graphical abstract

**Figure:**
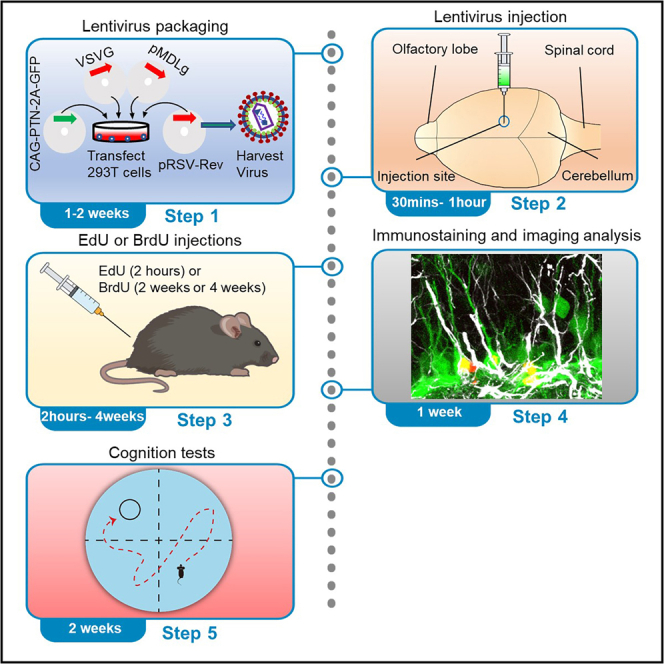

## Before you begin

Stereotactic lentiviral injection is a relatively rapid method to assess up- or down-regulation of different genes *in vivo*, which is widely applicated in the studies on neurodegenerative and neuropsychiatric disorders.^2^^,^^3^ Pleiotrophin (PTN), is a developmentally regulated and secreted growth factor, which has been identified that adult mouse hippocampal NSCs-derived PTN regulates newborn neuron development.^3^ To investigate whether autocrine PTN signaling could improve adult hippocampal neurogenesis, we delivered lentivirus expressing PTN into the DG of rodent hippocampus. Among all the experimental steps, placement of a mouse in a stereotactic frame is one of the most critical steps of this surgery. Majority of the issues happen here due to a loose head, or injury due to an excessive pressure. Therefore, it requires training and knowledge in how much the ear bars can be pushed towards the mouse skull. Here, this approach describes how to deliver lentiviruses (LVs) to target brain regions in rodents via stereotactic injection in detail.

***Note:*** Within each step of the protocol, if the recommended reagent/equipment is not available, you may try a product with similar functionality.

**CRITICAL:** It is highly recommended that people learn about vernier scales and safe mouse positioning before performing stereotactic injections.

### Institutional permissions

All experimental procedures in this protocol are carried out in accordance with the National Institutes of Health Guide for the Care and Use of Laboratory Animals and are approved by the Animal Care Committee and the Ethics Committee of the Third Affiliated Hospital of Sun Yat-sen University.

### Preparation of lentivirus packaging

**Timing: ∼3 days**

1. Extract the LVs target plasmid (CAG-PTN-2A-GFP, > 150 μg) and the three helper plasmids (VSVG, > 40 μg, pMDLg, > 100 μg and pRSV-Rev, > 55 μg).
2. Prepare adequate reagents: Dulbecco’s modified Eagle’s medium (DMEM, stored at 4°C for several month), Fetal Bovine Serum (FBS, stored at −20°C for several month), Penicillin streptomycin (PS, stored at −20°C for several month), Opti-MEM I Reduced Serum Media (stored at 4°C for several month), Polyethylenimine (PEI, 1 mg/mL, stored at 4°C for 12 month), Dulbecco’s phosphate-buffered saline (DPBS, stored at 25°C for several month).
3. Prepare culture HEK293T cells in 5 × 150 mm dishes with 13 mL culture medium (see materials and equipment section, stored at 4°C for up to 1 month) each at 37°C, 5% CO_2_.

***Note:*** You need to have 95%–100% confluent HEK293T in one 150 mm dish. Split 1 dish of 150 mm into 5 × 150 mm dishes (wash with DPBS and strong trypsin for 5 min). Each plate HEK293T cells should have 80% confluent at the moment of transfection.

### Preparation of lentivirus stereotactic injection

**Timing: ∼2 days**

4. Make sure to sterilize all surgical instruments (a pair of scissors, blunt-end forceps, a needle holder, scalpel, cotton swabs, absorbable sutures, and an electric razor) and experimental site (a stereotactic apparatus with digital display of the stereotaxic frame, a dental drill, a 5 μL Hamilton syringe fitted with a 33-gauge needle, a syringe pump controller and the rodent anesthesia machine).

***Note:*** Sterilize surgical instruments by autoclaving and sterilize the surgical field by UV irradiation.

5. Preparation of reagents (75% ethyl alcohol, isoflurane, analgesic (ketoprofen), erythromycin eye ointment and LVs).
6. Preparation of 12-month-old C57BL/6 male or female wild-type mice (ensuring that mice are of sufficient number, of the correct sex, and of suitable physical condition for the operation).

***Note:*** Adult C57BL/6 mice (12-month-old) are housed under standard conditions (feeding on a 12-h light/dark cycle with lights and given free access to food and water).

### Preparation for EdU or BrdU solution

**Timing: 1 h**

7. Prepare an EdU or BrdU solution at 10 mg/mL concentration.
  a. The solvent is DPBS.
  b. Dissolve by vortexing and heat in a 42°C thermometer if necessary.
  c. 10 mg EdU or BrdU dissolved in 1 mL DPBS will result in the applied concentration (10 mg/mL).

***Note:*** EdU and BrdU are recommended to be stored in powder form and protected from light at −20°C. Usually, fresh solution is required for application.

### Preparation of tissue and immunostaining solutions

**Timing: ∼2 days**

8. Preparation of 0.2 M phosphate buffer (PB, see materials and equipment section, stored at 25°C for up to 1 month), 4% paraformaldehyde (PFA, see materials and equipment section, stored at 4°C for up to 2 weeks protected from light), cryoprotectant solution (see materials and equipment, stored at 4°C for up to 1 month), Click Additive Solution (according to the manufacturer’s instructions (https://www.beyotime.com/product/ST067-50mg.htm) and store it at −20°C refrigerator after dispensing) and Polyvinyl alcohol (PVA)-DABCO solution(see materials and equipment, stored at −20°C for up to 6 months and 4°C for up to 1 week).

***Note:*** The components and storage methods are described in the Materials and Equipment section.

## Key resources table

**Table:** 

REAGENT or RESOURCE	SOURCE	IDENTIFIER
**Antibodies**		

Rat anti-BrdU (1:1,000)	Abcam	Cat# 6326; RRID: AB_305426
Rabbit anti-GFAP (1:1,000)	Cell Signaling Technology	Cat# 12389S
Mouse anti-DCX (1:200)	Santa Cruz Biotechnology	Cat# 271390
Rabbit anti-NeuN (1:500)	Abcam	Cat# 177487; RRID: AB_2532109
Goat anti-rabbit 647 (1:1,000)	Invitrogen	Cat# A-21245; RRID: AB_2535813
Goat anti-mouse 647 (1:1,000)	Invitrogen	Cat# A-21235; RRID: AB_2535804
Goat anti-rat 568 (1:1,000)	Invitrogen	Cat# A-11077; RRID: AB_2534121

**Bacterial and virus strains**		

DH5a	Tiangen	Cat#CB101-02
Trans110 chemically competent cell	TransGen Biotech	Cat#CD311-02

**Critical commercial assays**		

GoldHi EndoFree Plasmid Maxi Kit	CWBIO	Cat#CW2104M
EdU Cell Proliferation Kit	Beyotime	Cat#ST067

**Chemicals, peptides, and recombinant proteins**		

Goat serum	Gibco	Cat#16210072
Opti-MEM I reduced serum media	Gibco	Cat#31985070
DMEM/high-glucose medium	Wisent	Cat#319-005-CL
Dulbecco’s phosphate-buffered saline (DPBS)	Gibco	Cat#14190136
Phosphate-buffered saline (PBS)	Gibco	Cat#C10010500BT
L-glutamine	Gibco	Cat#25030081
Penicillin/streptomycin	Gibco	Cat#15140-122
Erythromycin eye ointment	Baiyunshan	Cat#212020050
Fetal bovine serum (FBS)	PAN-Seratech	Cat#ST30-3302
Polyethyleneimine (PEI)	Proteintech Group	Cat#PR40001
BrdU	Sigma	Cat#B9285
Isoflurane	RWD	Cat#R510-22-10
Ketoprofen	Santa Cruz	Cat#SC-363115Rx
Tris-HCl	Sigma	Cat#T3253
PVA-polyvinyl alcohol	Sigma	Cat#p8136
DABCO	Sigma	Cat#D2522
Sodium phosphate monobasic anhydrous (NaH_2_PO_4_)	Sigma	Cat#7558-80-7
Sodium phosphate dibasic anhydrous (Na_2_HPO_4_)	Sigma	Cat#71640
Paraformaldehyde (PFA)	Sigma	Cat#158127
Sodium hydroxide (NaOH)	Sigma	Cat#S8045
Glycerol	Fisher	Cat#G33-1
Ethylene glycol	Sigma	Cat#324558
0.9% Normal saline solution	Procell	Cat#PB180353
DAPI for nucleic acid staining	Sigma-Aldrich	Cat#D9542
Triton X-100	Beyotime	Cat#ST79

**Experimental models: Cell lines**		

HEK293T	ATCC	N/A

**Experimental models: Organisms/strains**		

Mouse: C57BL/6N, 12-month-old, males and females	Charles River	Strain code: 213

**Recombinant DNA**		

Plasmid: cPPT-CMV-eGFP-2A-PTN-WPRE (lentiviral vector)	This paper	N/A
Plasmid: pMDLg	Addgene	Cat#12251
Plasmid: pRSV-Rev	Addgene	Cat#12253
Plasmid: VSVG	Addgene	Cat#11912

**Software and algorithms**		

LAS X software	Leica Microsystems	N/A
SMART software V3.0	SMART Technologies	N/A
ImageJ	National Institutes of Health	RRID: SCR_002798; https://www.graphpad.com/
GraphPad Prism 9	GraphPad Software	RRID:SCR_002798; https://www.graphpad.com/

**Other**		

69100 Rotational digital stereotaxic instruments for mice and rat	RWD	Cat#K69100
KDS Legato 130 syringe pump	RWD	Cat#KDS LEGATO 130
Drill	RWD	Cat#78001
Rodent anesthesia machine	RWD	Cat#R500
Syringe holder	RWD	Cat#68218
1 mL syringe	KDL	Cat#60017031
1.5 mL microcentrifuge tubes	Axygen	Cat#MCT-500-C-S
50 mL centrifuge tubes	Corning	Cat#430291
75 cm^2^ culture flasks	Corning	Cat#430720
Fluorescence-activated cell sorting tube	STEMCELL	Cat#100-0087
Centrifuge tubes	Beckman Coulter	Cat#344058
Ultracentrifuge	Beckman Coulter	Cat#Optima XE-100 A94516
Animal platform	RWD	Cat#68607
Delicate scissor	RWD	Cat#S12003
Surgical handle	RWD	Cat#S32001
Delicate forceps	RWD	Cat#F12010
Haemostatic forceps	RWD	Cat#F21011
Needle holder	RWD	Cat#F31031
Heating pad	Nuansandong	Cat#CDT30X35
Suture	RWD	Cat#F35305-50
Suture needle	RWD	Cat#F35401-50
A drill bit	RWD	Cat#78041
Electric razor	BDL	Cat#K9
Microsyringe	Hamilton	Cat#87943
Microsyringe needle	Hamilton	Cat#780305
Gloves	Medicom	Cat#1154C
Masks	Medicom	Cat#2015M
Caps	Maydeal	Cat#60014533
Cotton swab	Winner	Cat#601-020764-01
150 mm dishes	Corning	Cat#430599
Vacuum filter/storage bottle system	Biosharp	Cat#BS-QT-037
Electronic balance	Yingheng	Cat#YHM-10002Y
Sliding microtome	Thermo Scientific	Cat#HM430

## Materials and equipment

**Table:** 

Culture medium		
Reagent	Final concentration	Amount
DMEM media	N/A	44.5 mL
FBS	10%	5 mL
PS	1%	0.5 mL
Total	N/A	50 mL

The media should be stored at 4°C for up to 3 weeks.

Fresh medium should be prepared before each differentiation experiment or kept sterile and stored at 4°C for up to 1 month.

**Table:** 

0.2 M PB		
Reagent	Final concentration	Amount
NaH_2_PO_4_ (MW 119.98)	N/A	5.52 g
Na_2_HPO_4_ (MW 141.96)	N/A	21.9 g
ddH_2_O	N/A	≈1000 mL
Total	N/A	1000 mL

The media should be stored at 25°C.

Fresh medium should be prepared or stored at 25°C for up to 1 month.

***Note:*** Dissolve first with 800 mL ddH_2_O and then add ddH_2_O until volume reaches 1 L. Adjust pH to 7.4. Pure the solution by filtering with a 0.22 μm filter.

**Table:** 

4% paraformaldehyde (PFA)		
Reagent	Final concentration	Amount
PFA	N/A	40 g
NaOH	1 M	1 mL
0.2 M PB	N/A	≈1000 mL
Total	N/A	1000 mL

PFA should be stored at 4°C and the other media should be stored at 25°C.

The medium should be stored at 4°C for up to 2 weeks protected from light.

***Note:*** Dissolve first with 800 mL 0.2 M PB and then add 0.2 M PB until volume reaches 1 L. Aliquot (50 mL is recommended), and store at −20°C.

**Table:** 

Cryoprotectant solution		
Reagent	Final concentration	Amount
Glycerol	N/A	50 mL
Ethylene glycol	N/A	50 mL
0.1 M PB	N/A	100 mL
Total	N/A	200 mL

The media should be stored at 25°C.

Fresh medium should be prepared or stored at 4°C for up to 1 month.

**Table:** 

Polyvinyl alcohol (PVA)-DABCO solution		
Reagent	Final concentration	Amount
Glycerin	N/A	6 g
PVA	N/A	2.4 g
ddH_2_O	N/A	6 mL
0.2 M Tris-HCl	N/A	12 mL
DABCO	N/A	0.625 g
Total	N/A	≈25 mL

The media should be stored at 25°C.

The medium should be stored at −20°C for up to 6 months and 4°C for up to 1 week.

***Note:*** Add 0.2 M Tris-HCl, adjust the solution to pH 8–8.5. Aliquot (1 mL is recommended) and store at −20°C. Do not refreeze.

## Step-by-step method details

### Production of lentivirus

**Timing: ∼1 week**

1. Check for the confluence of HEK293T cells (recommend is 80%) through the microscope and replace the culture medium with 10 mL fresh culture medium 1 h before transfection.

**CRITICAL:** If HEK293T cells have over-grown, showing lots of cluster and floaters, discard them and start new cells.

2. Prepare the mixture of helper plasmids and target plasmid with PEI (Figure 1).
  ***Note:*** Dilute total DNA (target plasmid and helper plasmid) and PEI (1 mg/mL) in 1:2.5 ratio (1 μg of DNA: 2.5 μL of PEI)
  a. Take one sterile 50 mL Eppendorf (EP) tube, add 5 mL of Opti-MEM I Reduced Serum Media, and add the plasmids as follows:

**Table:** 

Vector DNA	150 μg
VSVG	40 μg
pMDLg	100 μg
pRSV-Rev	55 μg
    Mix this mixture by pipette (approximately 10 times) using a 5 mL disposable pipette. Label the tube “DNA” and incubate for 5 min at 25°C.
    ***Note:*** When you first use those plasmids, check your plasmid with restriction enzymes or sequence to make sure you have right plasmid (e.g.: cutting the VSV-G plasmid with restriction endonucleases HindIII and EcoRI produces three fragments of 5.4 kb, 1.6 kb and 0.58 kb in length; cutting the pMDLg plasmid with restriction endonuclease EcoRI produces three fragments of 4.3 kb, 4.1 kb and 0.4 kb in length; and cutting the pRSV-Rev plasmid with restriction endonuclease EcoRI produces two fragments of 4.1 kb and 0.3 kb in length). The concentration of plasmid also needs to be confirmed to ensure the amount added (e.g.: the original concentration of VSV-G is 800 ng/μL (measured by NanoDrop instrument), therefore the volume of VSV-G added is 50 μL (40∗1000/800 = 50 μL)).
  b. Take another sterile 50 mL EP tube, add 5 mL of Opti-MEM I Reduced Serum Media, add 862.5 μL PEI (1 mg/mL), mix by pipette (approximately 10 times) using a 5 mL disposable pipette. Label the tube “PEI” and incubate for 5 min at 25°C.
    ***Note:*** PEI, a non-viral cationic polymer, can be used to transfect plasmid DNA or siRNA into suspension or adherent cells due to its low toxicity, low cost, and low immunogenicity.^4^
    ***Alternatives:*** You can use Lipofectamine 3000 transfection reagent to replace PEI.^5^
  c. Add PEI diluent to plasmid diluent 50 mL EP tube (DNA tube), gently mix by pipette (approximately 10 times) using a 10 mL disposable pipette. Incubate for 20 min at 25°C.
  d. Take out 5 dishes of HEK293T cell from incubator, add 2 mL the mixture of DNA-PEI drop by drop to each dish, gently mix, mark dishes, and put them back to incubator.

**Figure 1:**
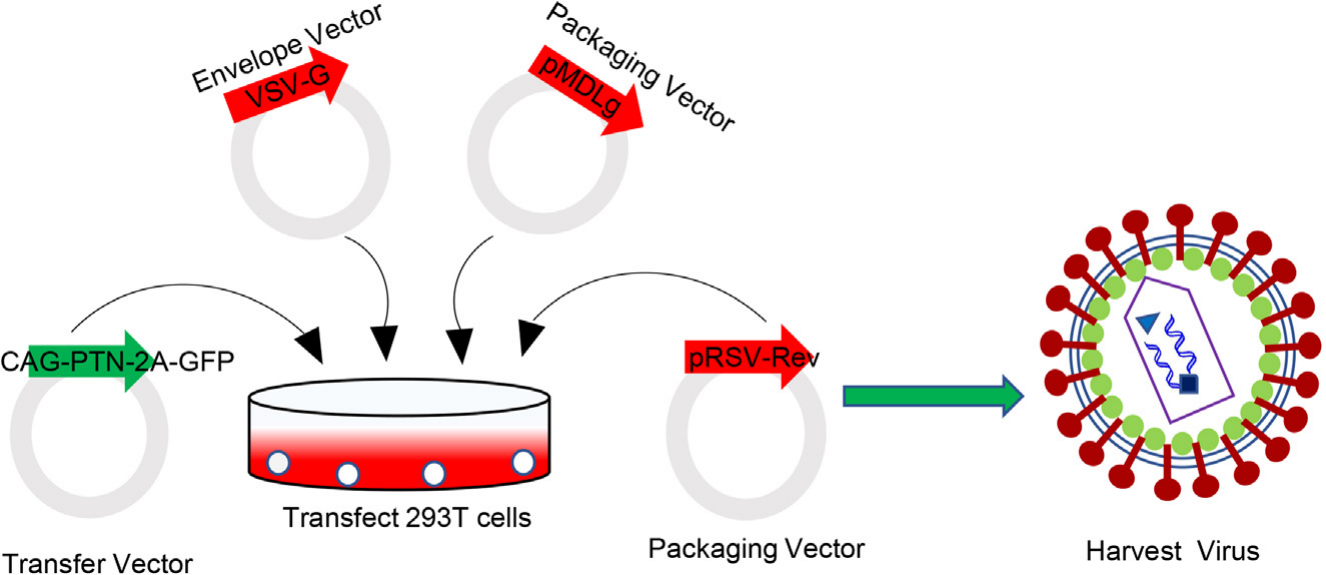
Schematic of production of lentivirus (LVs) from transfected HEK293T cells LVs that up-regulation of PTN gene were constructed by the third-generation lentiviral vectors. Virus particles are harvested immediately after consecutive 3-day viral supernatant collection.
3. Remove media by aspiration after 4–6 h transfection, add 13 mL fresh culture media (see materials and equipment) to each dish.

**CRITICAL:** Transfection time cannot over 6 h, if the transfection time is too long, it will affect the cell heath and survival.

4. Harvest released viral particles by collecting viral supernatant at 40, 64, and 88 h in a T75 flask, stored at 4°C.

***Note:*** It is important to change the media with extreme gentleness to avoid the detach from the plate.

**CRITICAL:** Check the transfection efficiency approximately 40 h after transfection through detecting whether the transfected cells have bright florescent. If efficiency is <80%, it means that you should have not made virus out of it, stop your experiment.

**Figure 2:**
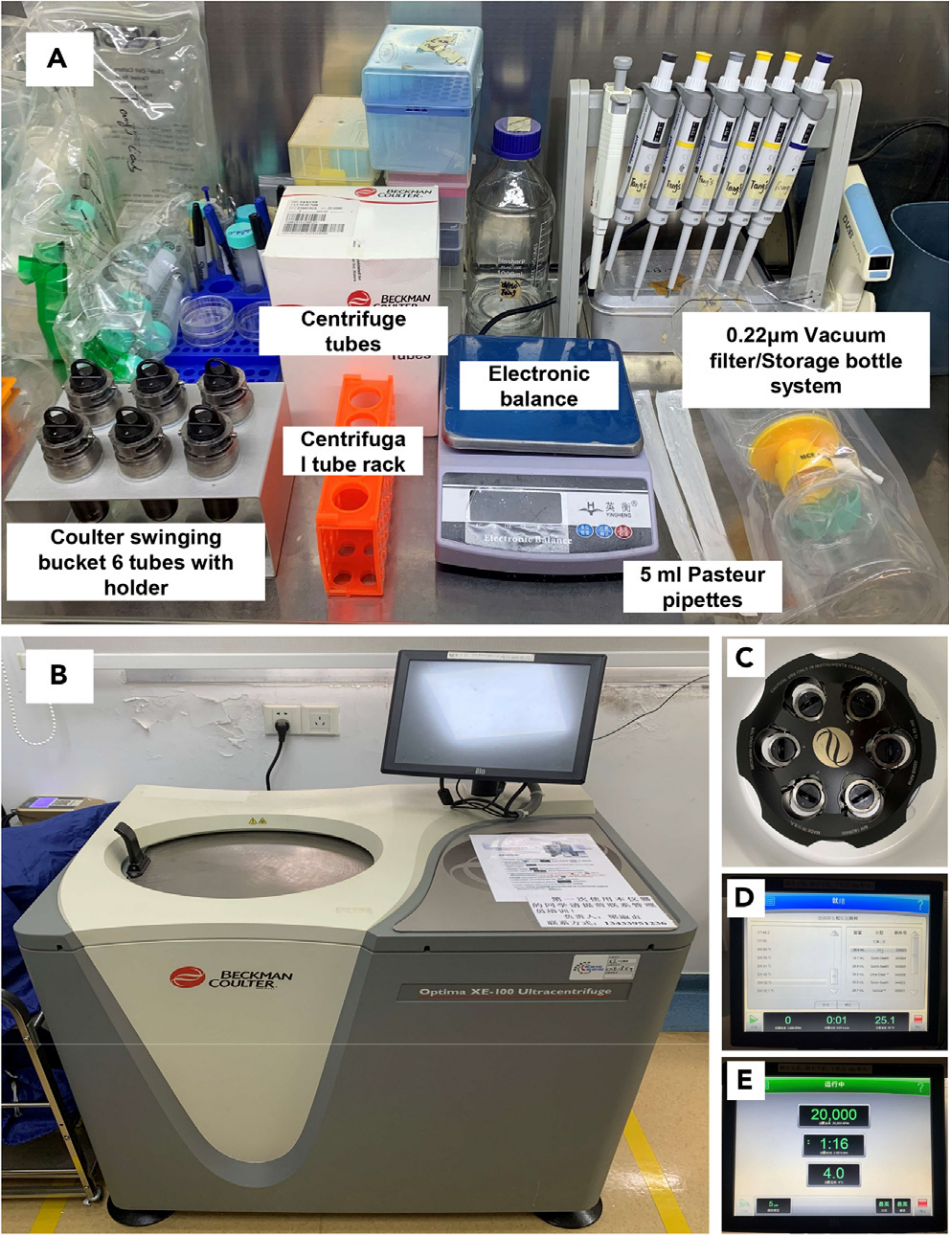
Preparation for lentivirus (LVs) harvest by ultracentrifugation (A) Main components before LVs harvest by ultracentrifugation. (B) The ultracentrifuge is used for LVs harvest. (C) The proper SW32Ti swinging-bucket rotor and the matched six tubes were well installed. (D) Choose the right rotor and laboratory consumables. (E) Set up the correct parameters in order: 20,000 rpm, 2 h, and 4°C.

5. Filter virus through a 0.22 μm vacuum bottle filter and centrifuge at 49000 g, 4°C for 2 h. Discard the supernatant by aspiration (Figure 2).

***Alternatives:*** If you do not have ultracentrifuge, you can use polyethylene glycol 6000 to concentration and purification of the virus.^6^

6. Wash virus once with cold DPBS and then perform the final centrifugation at 49000 g, 4°C for 2 h.
7. Leave approximately 100 μL of cold DPBS in the tube to resuspend the viruses’ particles, and then stored at 4°C for 24 h.
8. Aliquot the viruses at 10 μL per 1.5 mL tube, store at −80°C.

***Note:*** All instruments, container, tips, tubes used for packaging LVs should be decontaminated with freshly made 10% bleach after use.

9. Measure virus titer by infecting HEK293T cells with serially diluted virus preparations.
  a. Plate HEK293T cells in 6-well plate (5 × 10ˆ4 cells/per well) a day before viral titer infection.
  b. Prepare viruses of different titers by serial dilution (1:1, 1:10, 1:100…1:10ˆ6).
  c. 2 μL different titers of virus are added separately to each well with 72–120 h incubation at 37°C.
  d. Wash with PBS three times.
  e. Add 200 μL trypsin (stored at 4°C for several months) to each well for 5 min, then collect the cells solution into a fluorescence-activated cell sorting (FACS) tube and add 600 μL 4% PFA.
    ***Note:*** Samples can keep stable for one week when stored at 4°C in the dark.
  f. Calculate viral titer from FACS result.

Since 5∗10ˆ4 cells are cultured the day before infection, there are 1∗10ˆ6 cells at the time of viral infection. It is calculated as follows.

**Table:** 

1 μL (1:1)	75%	(It should be 100%, but often isn’t)
0.1 μL (1:10)	25%	1∗10ˆ6 ∗ 25% /0.1 = 2.5∗10ˆ5/μL
0.01 μL (1:100)	4.5%	1∗10ˆ6 ∗ 4.5%/ 0.01 = 4.5∗10ˆ5/μL
0.001 μL (1:1000)	0.5%	1∗10ˆ6 ∗ 0.5% /0 .001 = 5.0∗10ˆ5/μL

The average of the three viral concentrations is (2.5 + 3.5+5)/3∗10ˆ5 = 4.0∗10ˆ5 particles/μL or 4.0∗10ˆ8 particles/mL. The viral titer is 4.0∗10ˆ8 particles/mL.

***Note:*** A minimum of ∼1×10^9^ infectious particles per ml is recommended.

For more detailed protocol of lentivirus production see Fricano-Kugler et al. and Guo et al.^6^^,^^7^

### Preparation for surgery of animals

**Timing: 1 h**

**Figure 3:**
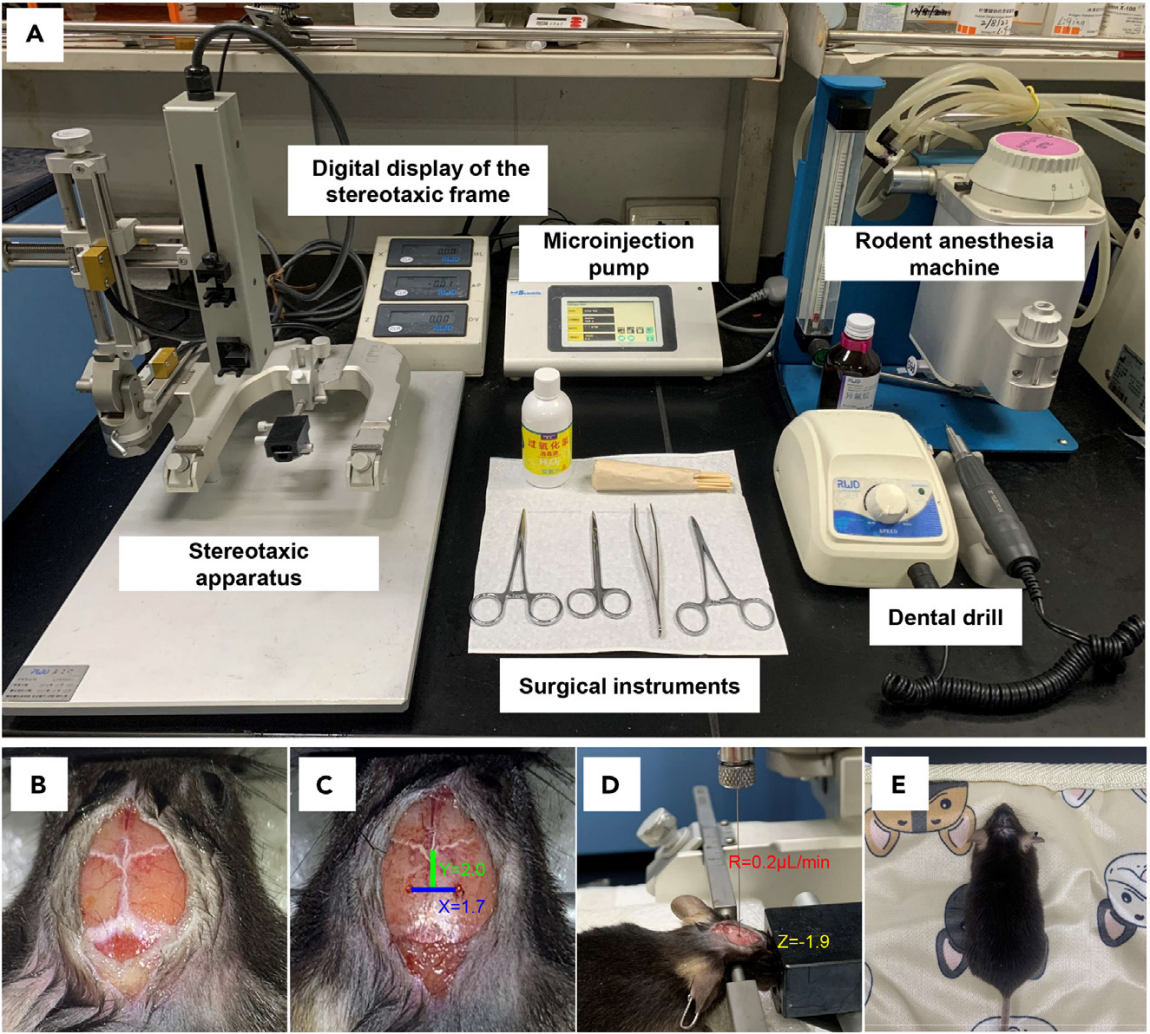
Surgical instruments and key procedures of lentivirus (LVs) stereotactic injection into the dentate gyrus (DG) of adult hippocampus (A) Main components of the stereotactic system. (B) Stereotaxic markers on the skull. (C) Two small holes drilled for LVs injection. The coordinate used for the LVs injection into the DG (AP: 2.0 mm, ML: ±1.7 mm, DV: -1.9 mm). (D) LVs injection at a rate of 0.2 μL/min through the syringe pump controller. (E) After injection, close the incision and place the mouse on a heating pad until it wakes up.
11. Anesthetize the mouse with isoflurane by a mask.

***Note:*** Induction with 5% gas in air mixture before transferring to the stereotactic frame, then maintenance at 2%.

10. Assemble the stereotaxic instrument, the dental drill and the rodent anesthesia machine (Figure 3A).

***Alternatives:*** The 2% isoflurane can be replaced by sodium pentobarbital (50 mg/kg, intraperitoneal (i.p.) injection) or avertin (400 mg/kg i.p. injection).

12. Wait until the mouse is fully anesthetized, then inject Ketoprofen (3–5 mg/kg) via i.p. route to manage pain.

***Alternatives:*** Ketoprofen can be replaced by Carprofen (5–10 mg/kg, subcutaneous injection).

13. Place the mouse on a heating pad to keep a steady body temperature.

***Note:*** Mice under anesthesia may have difficulties regulating body temperature, a heat pad will prevent hypothermia and unexpected animal deaths during surgical procedures.

14. Moisten the mouse eyes with ointment to prevent drying and infection.
15. Use an electric razor to remove hair from the top of the head.
16. Sanitize the skin with 75% ethanol.

***Note:*** If possible, please perform a complete surgical scrub consisting on 3 independent cleaning procedures alternating Alcohol and iodine.

### Exposure of the skull and drilling

**Timing: 10 min**

17. Latch the mouse front teeth onto the anterior clamp of the adapter on the stereotactic instrument, and adjust the rods into the crevices just anterior to the animal’s ears to fix the head.

***Note:*** Make sure the mouse head well fixed and firm.

18. Use a scalpel to make an anterior-posterior incision of about 1.5 cm between the ears.

***Note:*** A local injection of 0.25% bupivacaine (30 μL) for at least one minute prior to incision can help improve postoperative pain until the NSAIDs kick in.

19. Using a sterile cotton swab clean the surface of the skull to make sure the bregma and the lambda are visible (Figure 3B).

***Note:*** Bregma is the intersection between the coronal and sagittal sutures; Lambda is the intersection between the coronal and lambdoid sutures.

20. Point the tip of the microinjection syringe, held by the needle holder, to the bregma point, considered as the zero point of the three axes.
21. Keep the syringe on a vertical axis so as not to scratch the skull during planar movement and then move the syringe head to the correct position.

**CRITICAL:** Zero the instrument on the range to be used for measurement.

***Note:*** Not all stereotactic frames are built in digital measure tools. It requires operators have a knowledge in the use of Vernier scale in non-digital equipment.

22. Find the coordinates of the DG based on the atlas of the mouse brain^8^: −2.0 mm posterior to the bregma, ±1.7 mm lateral to the midline, -1.9 mm ventral to the surface of the skull.
23. Move the needle of the syringe according above coordinates, label the two positions with a marker.
24. Raise the syringe in the vertical axis and move the tip to a safe position.
25. Using a fine dental drill (0.6 mm drill bit) carefully grind the skull at the target site to two shallow holes (Figure 3C).

***Note:*** It is recommended to use angled Drill position instead of vertical to avoid falling deep in to the brain tissue. If bleeding occurs during this procedure, use a small medical cotton ball to soak up the blood.

**CRITICAL:** Drilling holes must be properly controlled, otherwise brain tissue damage can easily be caused accidentally after drilling through the skull, which adversely affects the process and results of the experiments.

### Lentivirus injection

**Timing: ∼20 min**

26. Take out the moderate consumption of LVs from -80°C refrigerator, and put it on the ice right before viral loading.

***Note:*** Lentivirus expressing PTN are named as lenti-PTN and lentivirus without the gene of interest (e.g.: PTN) are named as lenti-NC to be prepared and used as control.

**CRITICAL:** Virus is stored in the freezer at −80°C. Use it immediately after removal, and avoid multiple freeze-thaw processes.

27. Clean the microinjection syringe with sterile PBS and withdraw 1 μL of the concentrated LVs solution.

***Note:*** When connecting the needle to the micro syringe pump, ensure that the tip of the needle is vertical and that there are no air bubbles inside the needle. A good practice is to collect and withdraw volumes to remove the air. Specifically, a first collection with a Hamilton will take significant air that can be seen in the plunger end (more than 5μL).

28. Place the syringe over the hole and slowly lower it vertically until it reaches the surface of the brain. Set the dorsal/ventral (D/V) coordinate (depth) to 0.
29. Lower the syringe to reach the DG, set D/V to −1.9 mm (Figure 3D).
30. Set the digital pump to 0.2 μL/min (1 μL would be injected in 5 min) and start infusion.

***Note:*** Infusion at a slow pace allows the virus to spread efficiently into the tissues and prevents reflux.

31. After finishing the infusion, wait an additional 5 min to complete diffusion of LVs into the brain rather than backflow of viral solution up the needle track.
32. Slowly remove the syringe out of the brain and watch for fluid backflow.

***Note:*** If reflux is clearly observed, it means partial loss of LVs injection.

33. Rinse the microinjection syringe with sterile PBS again to remove traces of blood on the tip of the needle and keep the syringe is unobstructed.
34. Repeat step 27–32 for another hemisphere.

For more detailed protocol of LVs injection, see Li et al. and Tang et al.^1^^,^^3^

### Wound sealing and post-operative care

**Timing: 30 min to 1 h**

35. Suture the wound with absorbable thread.
36. Gently remove the ear bars from the stereotaxic frame and take the mouse from the operating table.
37. Place the mouse in a new clean cage and keep it on a heating pad (Figure 3E).
38. Closely monitor the mouse until it wakes up and moves on its own.
39. Return the mouse to the standard housing condition.

***Note:*** Mice are required in recovery for 72 h before undergoing the next experimental step.

### BrdU injection

**Timing: 4 days**

3 days after the operation, mice receive BrdU injection to assess NSCs differentiation.

**Figure 4:**
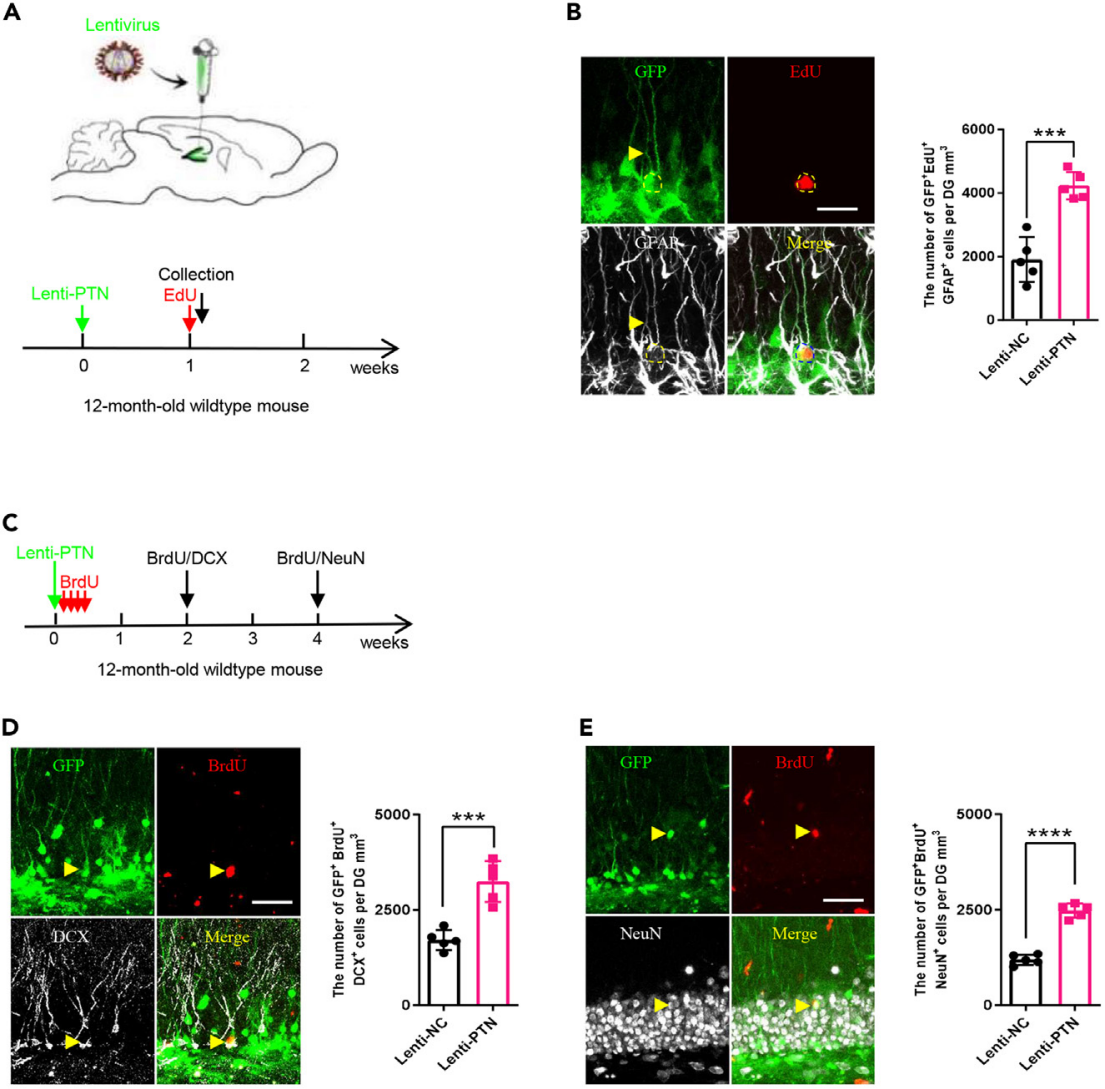
PTN promote NSCs proliferation and differentiation via lentivirus (LVs)-mediate overexpression (A) Schematic diagram and timeline for investigating the LVs -mediated PTN overexpression effect on NSC proliferation *in vivo* of 12-month-old wild-type mice. (B) Representative images (left) and quantification (right) of GFP^+^ (green) EdU^+^ (red) GFAP^+^ (silver) proliferating NSCs in the DGs between the two groups mice. Scale bars, 50 μm, n = 5 mice per group. (C) Timeline for investigating the LVs -mediated PTN overexpression effect on NSC differentiation *in vivo* of 12-month-old wild-type mice. (D) Representative images (left) and quantification (right) of GFP^+^ (green) BrdU^+^ (red) DCX^+^ (silver) immature neurons in the DGs between the two groups of mice. Scale bars, 50 μm, n = 5 mice per group. (E) Representative images (left) and quantification (right) of GFP^+^ (green) BrdU^+^ (red) NeuN^+^ (silver) mature differentiated neurons in the DGs between the two groups mice. Scale bars, 50 μm, n = 5 mice per group. All data are presented as the mean ± SEM. Statistical evaluation was performed with Student’s t test. Nonsignificant comparisons were not identified. ∗p < 0.05, ∗∗p < 0.01, ∗∗∗p < 0.001, ∗∗∗∗p < 0.0001.

40. Treat mice with BrdU at 50 mg/kg i.p. for 4 days and sacrificed at 2 or 4 weeks after injection to assess NSCs differentiation *in vivo* (Figure 4C).

**CRITICAL:** Make fresh solution for BrdU injection each day.

***Note:*** Take the injection at the same time each day.

### EdU injection

One week after the operation, mice receive EdU injection to assess NSCs proliferation.

***Note:*** In this study, PTN overexpressing lentivirus showed higher infection efficiency in mouse hippocampus one week after injection, so we performed proliferation assay one week later. However, the timing can be adjusted depending on the purpose of the experiment; for example, some studies have assayed NSCs proliferation 2 or 3 weeks after viral injection.^7^^,^^9^

41. Treat mice with EdU at 100 mg/kg i.p. and then sacrificed 2 h after injection to assess NSCs proliferation *in vivo* (Figure 4A).

**CRITICAL:** Make fresh solution for injection.

### Brain sections preparation

**Timing: ∼4 days**

***Note:*** At the experimental time point, mouse is anesthetized and transcardially perfused with 0.01 M PBS followed by 4% PFA.^3^^,^^10^

42. Fix brain in 4% PFA for 24 h.
43. Equilibrated in 30% sucrose (stored at 4°C for 1 month) for 2 days.
44. Cut 40 μm-thick coronal sections by a sliding microtome.
45. Floating sections stored in 96-well plates filled with cryoprotectant solution at 4°C.

### EdU detection and immunofluorescent staining

**Timing: 2 days**

46. Take out brain sections of 96 well plates.
47. Wash the floating sections with 0.01 M PBS three times, each time for 5 min at 25°C, and gently shaking.
48. Incubate the floating sections in PBS- 0.5% Triton (stored at 4°C for 1 week) for 20 min at 25°C.
49. Repeat the step 47.
50. Prepare the Click Reaction Solution according to the manufacturer’s instructions (https://www.beyotime.com/product/ST067-50mg.htm)

***Note:*** Click Reaction Solution (500 μL) containing:

**Table:** 

Click reaction buffer	430 μL
CuSO_4_	20 μL
Azide 568	1 μL
Click Addictive Solution	50 μL

Prepare the Click Reaction Solution strictly in the order and the volume of above components, otherwise the reaction may not be carried out efficiently.

**CRITICAL:** Click Reaction Solution must be used within 15 minutes of preparation.

51. Remove the PBS and incubate the sections with proper Click Reaction Solution (500 μL per well/24-well plates) at 25°C for 30 min.

***Note:*** From the step 51, the brain sections should be protected from light.

52. Repeat the step 47.
53. Blocking: remove PBS and add blocking buffer (containing 300 μL/10 mL goat serum and 250 μL/10 mL 10% Triton X-100, stored at 4°C for 1 week)
54. Incubate at 25°C for 1 h while gently shaking.
55. Remove the blocking buffer and add primary antibodies such as Rabbit anti-GFAP, diluted in blocking buffer (1:1,000).
56. Incubate at 4°C for 24 h while gently shaking.
57. Repeat the step 47.
58. Remove the PBS and add secondary antibodies diluted in PBS (1:1,000).
59. Incubate at 25°C for 1–2 h while gently shaking.
60. Repeat the step 47.
61. Mount with PVA-DABCO solution and coverslip with appropriately sized glass covers.
62. Let dry completely before imaging.

For more detailed protocol of immunostaining, see Li et al., Tang et al., and Zhao and van Praag.^1^^,^^3^^,^^11^

### BrdU detection and immunofluorescent staining

**Timing: 2 days**

63. Repeat the step 46–47.
64. Incubate in 2 M HCl (stored at 25°C for several month) at 37°C for 20 min using water-bath.

***Note:*** HCl should be pre-warmed.

65. Incubate in 0.1 M Borate buffer (PH = 8.0, stored at 25°C for several month) for 20 min on a shaker at 25°C.

***Note:*** Try to remove HCl completely before rinsing in Borate using the Pasteur pipet.

66. Repeat the step 52–54.
67. Remove the blocking buffer and add primary antibodies such as Rat anti-BrdU (1:1,000) and Mouse anti DCX (1:200) or Rabbit anti NeuN (1:500), diluted in blocking buffer.
68. Repeat the step 56–62.

### Behavioral assessment

**Timing: ∼2 weeks**

The behavioral assessment in the Morris Water Maze (MWM) and the Reverse Morris Water Maze (RMWM) are performed after 4 weeks, at which LVs usually infect most cells within the injection sphere. These behavior tests reflect spatial learning and memory ability of mice. The detailed protocol of MWM and RMWM see Vorhees and Williams and Chen et al.^12^^,^^13^

***Note:*** The RMWM test was conducted 48 h after the probe test of the MWM test.

## Expected outcomes

**Figure 5:**
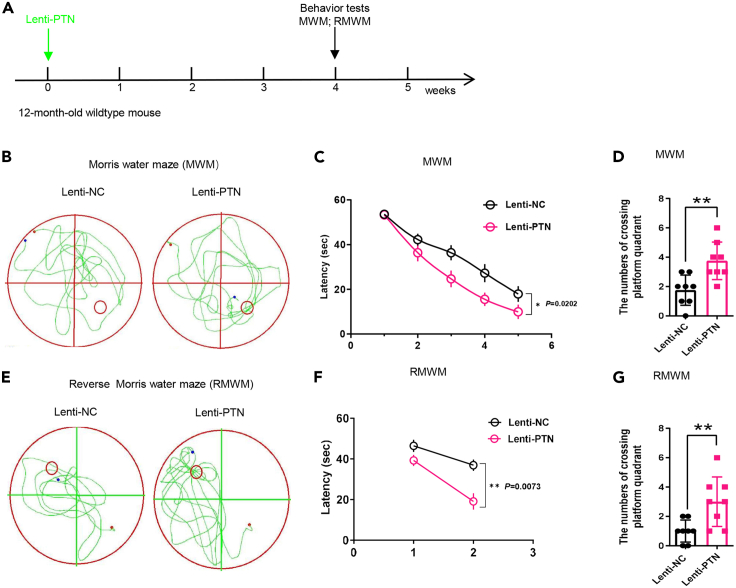
PTN ameliorates age-induced cognitive impairments (A) Timeline for the MWM and RMWM behavioral tests in 12-month-old mice with PTN overexpression by stereotaxic injection of Lenti-PTN. (B) Representative MWM movement paths of the two groups mice. (C) Quantification of MWM escape latencies to find the platform. n = 8 mice per group. (D) Quantification of MWM platform quadrant crossing numbers. n = 8 mice per group. (E) Representative RMWM movement paths of the two groups mice. (F) Quantification of RMWM escape latencies to find the platform. n = 8 mice per group. (G) Quantification of RMWM platform quadrant crossing numbers. n = 8 mice per group. All data are presented as the mean ± SEM. Statistical evaluation was performed with Two-way ANOVA and Tukey’s post hoc multiple comparisons for (C) and (E), and Student’s t test for (D) and (G). Nonsignificant comparisons were not identified. ∗p < 0.05, ∗∗p < 0.01, ∗∗∗p < 0.001, ∗∗∗∗p < 0.0001.

To investigate whether pleiotrophin (PTN) affected proliferation and differentiation of NSCs, we stereotaxically injected LVs co-expressing green fluorescent protein (GFP) with PTN into the DG of hippocampus to induce PTN overexpression of the old mouse (12-month-old wild-type mouse) (Figure 4A). After one week LVs injection, we treated 5 mice of each group (PTN group and the control group) with EdU by i.p. injection 2 h before sacrificing to label proliferating cells (Figure 4A). As expected, PTN overexpression in the DG of the old mouse resulted in a significant increase in the number of GFP^+^EdU^+^GFAP^+^ NSCs (with a typical NSC morphology) (Figure 4B), which suggests that PTN promotes the proliferation of NSCs. And we treated another cohort mice of the two groups with BrdU through i.p. injection for 4 days and sacrificed at 2 or 4 weeks after injection respectively to label immature neurons and mature neurons *in vivo* (Figure 4C). Similarly, the numbers of both GFP^+^BrdU^+^DCX^+^ immature neurons (Figure 4D) and GFP^+^BrdU^+^NeuN^+^ mature differentiated neurons (Figure 4E) were remarkably increased, suggesting that PTN induces more neuronal differentiation. Moreover, we subjected new cohort mice of the two groups to perform MWM and RMWM tests 4 weeks after injection lentiviruses to assess their cognitive function (Figure 5A). We found that either in MWM or RMWM tests, PTN group mice crossed the target quadrant more times and spent less time in the latency than the control group mice (Figures 5B–5G), indicating better spatial memory. Taken together, these results demonstrated that PTN ameliorates both age-induced hippocampal neurogenesis defects and cognitive dysfunction.

## Limitations

We have evaluated transfection efficiency by detecting native fluorescence protein of infected cells. Although it is a simple and straightforward method, one possible technical limitation is transfected cells might exhibit lower sensitivity of native fluorescence detection due to cell types and transfection time. Thus, immunohistochemistry and subsequent indirect fluorescence detection is more credible.

The viral vector in our study is popular and has been used in our previous studies.^3^

Because each viral vector varies in dissemination capacity, packaging capacity, tropism, transgene expression time, and expression duration. Moreover, it is challenging, particularly in brain *in vivo*, since the quantity of infected cells in the specific tissue can be limiting. If possible, pilot studies should be performed before the formal experiment to determine the dosage of virus and validate viral function.

## Troubleshooting

### Problem 1

Transfection efficiency is < 80% or the transfected cells are not bright fluorescent (step 1–4).

### Potential solution

• Check whether the plasmid and transfection reagent are used correctly.
• Ensure plasmids are propagated in recombination-deficient *E.coli.*
• Check quality and concentration of plasmid. Make sure plasmid concentrations are > 1 μg/ μL and all reagents are functioning properly.
• Check HEK293T cells every day whether are healthy, split on a regular schedule, and never overgrown (more than 80% confluence).
• Change a stronger promoter to drive the fluorescent protein expression.

### Problem 2

Failure on stereotaxic injection of hippocampus (step 17–29).

### Potential solution

• Ensure that the stereotactic frame and accessories (including ear bars and incisor adapters) are appropriate for the type of animal undergoing surgery.
• Make sure the animal reaches a sufficient level of surgical anesthesia, showing as mice in supine position with uniform heartbeat and respiration, muscle relaxation, no movement of limbs, no touch response of whiskers, and loss of pedal reflexes.
• Keep the head securely placed and well-fixed in the stereotaxic instrument.
• Use hydrogen peroxide solution to ensure bregma and lambda are clearly visible, which is required for accurate coordinates.

### Problem 3

Too much solution was left in microinjection syringe (Step 30–34).

### Potential solution

• LVs are used immediately once removed and quickly returned to ice after usage.
• Slowly and evenly inject the LVs into the DG to prevent backflow of viral solution.
• Left the syringe tip in place for 5 min after delivering virus. During this period, the syringe is slightly retracted along the Z-axis (<1 mm) to disperse the viral solution in the tissues more homogeneously.^2^

### Problem 4

Failure on EdU detection (Step 46–62).

### Potential solution

• Ensure mice are intraperitoneal injected with EdU at 100 mg/kg, 2–4 h prior to sacrifice.
• Check all reagents are functional.
• Ensure that the Click reaction solution is freshly prepared and use it within 15 min.
• Be sure to protect samples from light since adding Click reaction buffer.
• Avoid selecting secondary antibodies with the same fluorescent color as Azide.

### Problem 5

Brain slices move during mounting and imaging (Step 61–62).

### Potential solution

• Gently transfer brain slices on the glass slide and dry the surrounding solution completely with a blow dryer if necessary.
• Use adequate amount of PVA-DABCO solution.
• Before imaging, ensure the PVA-DABCO solution is stable.
• Wipe excess PVA-DABCO solution off the coverslip as gently as possible with anhydrous ethanol.

## Resource availability

### Lead contact

Further information and requests for resources and reagents should be directed to and will be fulfilled by the lead contact, Changyong Tang (tangchy23@mail.sysu.edu.cn).

### Materials availability

Plasmids used in this protocol are available under request to the lead contact, Changyong Tang.

### Data and code availability

This study did not generate new unique data or code.
